# Supplementary material for: Infant HIV-protection: Comparing antiretroviral therapy, maternal and infant factors influence on infant HIV acquisition in Uganda: A six-year real-world experience
Source: PLOS Glob Public Health. 2026 Feb 13;6(2):e0004896. doi: 10.1371/journal.pgph.0004896 (PMC12904416; doi:10.1371/journal.pgph.0004896)
Supplement: S1 Fig — (DOCX) [file pgph.0004896.s001.docx]

**Impact of Negative Maternal and Infant Factors on Infant HIV Positive Outcome**

Among infants with positive outcomes, maternal age and feeding practice appeared to have a stronger influence than viral suppression alone. While viral suppression generally aligned with favorable outcomes, positive cases were more frequent among mothers aged ≥35 years, particularly those practicing mixed or unknown breastfeeding. In contrast, most HIV-negative infants were born to virally suppressed mothers aged 25–34 years who exclusively breastfed. Unsuppressed mothers contributed minimally to negative outcomes, reaffirming that suppression remains protective. Overall, behavioral and sociodemographic factors linked to age and feeding choice may have had a greater impact on HIV positive infant outcomes, suggesting these factors were more influential than maternal viral suppression, which played a contributory but not dominant role.

**S1 Fig: Impact of Negative Maternal and Infant Factors on Infant HIV Positive Outcome**


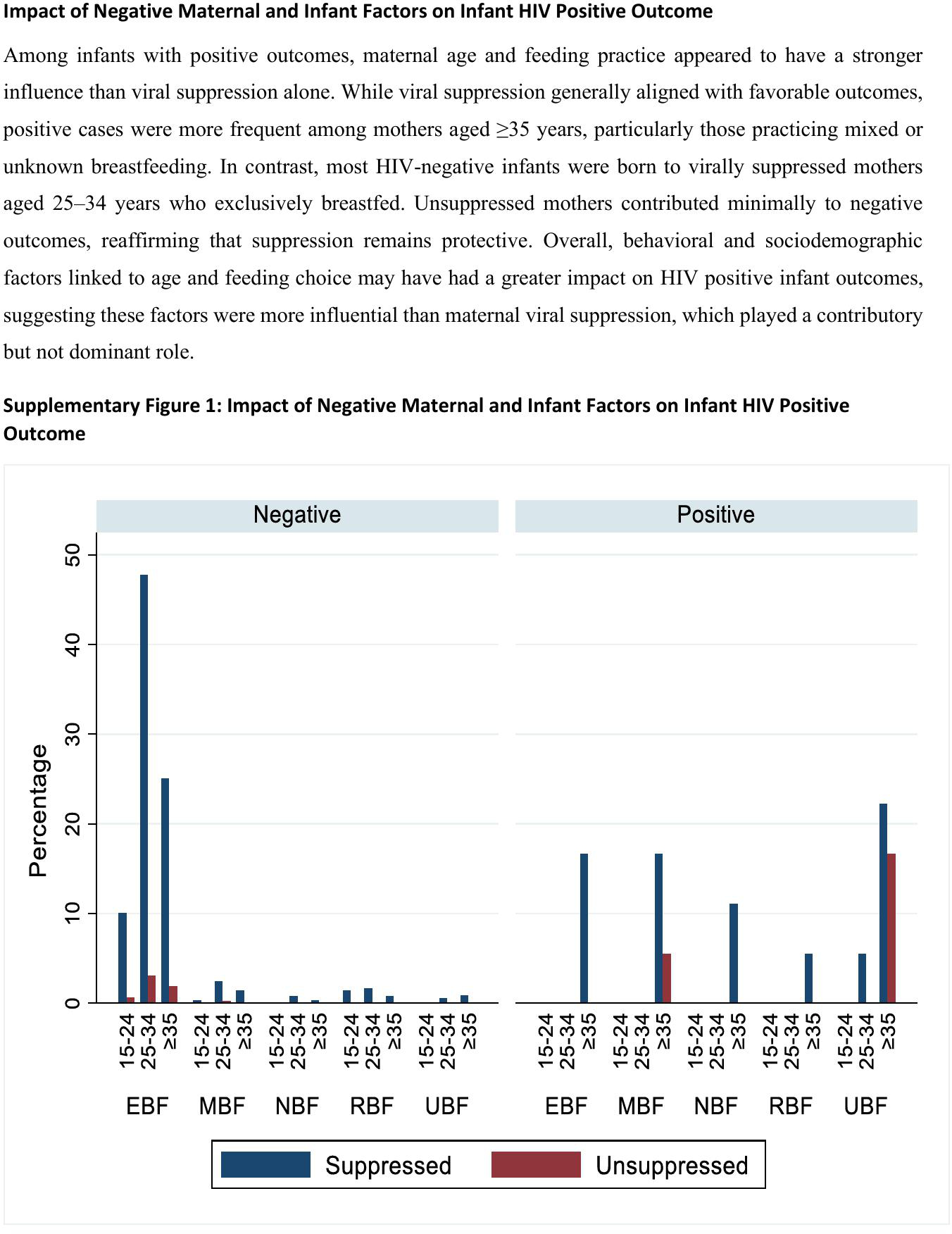


Note **Infant** Feeding categories s are as follows: EBF: Exclusive breastfeeding, MBF: Mixed breastfeeding, NBF: No breastfeeding, RBF: Replacement feeding, UBF: Unknown breastfeeding
