# Supplementary material for: Infant HIV-protection: Comparing antiretroviral therapy, maternal and infant factors influence on infant HIV acquisition in Uganda: A six-year real-world experience
Source: PLOS Glob Public Health. 2026 Feb 13;6(2):e0004896. doi: 10.1371/journal.pgph.0004896 (PMC12904416; doi:10.1371/journal.pgph.0004896)
Supplement: S1 Table — (DOCX) [file pgph.0004896.s003.docx]

**S1 Table: Showing time of positive HIV diagnosis among infants**

| **Time of Diagnosis** | **Positive Outcomes** | **Proportion (%)** | **95% Confidence Interval** |
| --- | --- | --- | --- |
| 0-2 months | 25 | 59.5 | 44.5–73.0 |
| 9 months | 2 | 4.8 | 1.3–15.8 |
| 12-15 months | 12 | 28.6 | 17.2–43.6 |
| 18-24 months | 3 | 7.1 | 2.5–19.0 |
| **Total** | 42 | 100 | — |
