# Supplementary material for: Infant HIV-protection: Comparing antiretroviral therapy, maternal and infant factors influence on infant HIV acquisition in Uganda: A six-year real-world experience
Source: PLOS Glob Public Health. 2026 Feb 13;6(2):e0004896. doi: 10.1371/journal.pgph.0004896 (PMC12904416; doi:10.1371/journal.pgph.0004896)
Supplement: S2 Table — (DOCX) [file pgph.0004896.s004.docx]

**S2 Table: Maternal Characteristics for positive infants**

| **Variable** | **Categories** | **Proportion of HIV positive infants**  **n (%)** |
| --- | --- | --- |
| Age (Years) | 15-24 | 0 (0) |
|  | 25-34 | 1 (5.6) |
|  | >35 | 17 (94.4) |
| WHO stage | Stage 1 | 18 (100) |
|  | Stage 2 | 0 (0) |
|  | Stage 3 | 0 (0) |
|  | Stage 4 | 0 (0) |
| Adherence | Good | 17 (94.4) |
|  | Fair | 0 (0) |
|  | Poor | 1 (5.6) |
| Start Regimen | DTG | 2 (11.1) |
|  | EFV | 11 (61.1) |
|  | NVP | 3 (16.7) |
|  | PI | 1 (5.6) |
| Drug line | First line | 16 (88.9) |
|  | Second line | 2 (11.1) |
|  | Third line | 0 (0) |
| Duration on ART (years) | >1 | 2 (11.1) |
|  | 1-2 | 2 (11.1) |
|  | >2-5 | 6 (33.3) |
|  | >5 | 8 (44.4) |
| History of regimen change | No | 2 (11.1) |
|  | Yes | 16 (88.9) |
| ART experience | Experienced | 16 (88.9) |
|  | Naive | 2 (11.1) |
| Viral load suppression | Suppressed | 14 (77.8) |
|  | Unsuppressed | 4 (22.2) |
| Marital status | Married | 10 (55.6) |
|  | Single | 5 (27.8) |
|  | Separated/Divorced/Widowed | 2 (11.1) |
|  | Unknown | 1 (5.6) |
| Mode of delivery | Caesarean section | 5 (27.8) |
|  | SVD | 13 (72.2) |
| Place of delivery | Health facility | 18 (100) |
|  | Home/ Unknown | 0 (0) |
| History of ART transition* | 0 | 5 (27.8) |
|  | 1 | 0 (0) |
|  | 2 | 1 (5.6) |
|  | 3 | 0 (0) |
|  | 4 | 2 (11.1) |
|  | 5 | 8 (44.4) |
|  | 6 | 1 (5.6) |
|  | 7 | 0(0) |
| *The study categorized antiretroviral therapy (ART) exposure based on maternal regimen transitions during pregnancy and breastfeeding. These included: transition from other regimens to DTG-based (0), EFV-based (1), PI-based (2), or NVP-based (3) regimens, as well as cases where PWLH remained on the same regimen throughout; DTG-based (4), EFV-based (5), PI-based (6), or NVP-based (7). | | |
