# Supplementary material for: Infant HIV-protection: Comparing antiretroviral therapy, maternal and infant factors influence on infant HIV acquisition in Uganda: A six-year real-world experience
Source: PLOS Glob Public Health. 2026 Feb 13;6(2):e0004896. doi: 10.1371/journal.pgph.0004896 (PMC12904416; doi:10.1371/journal.pgph.0004896)
Supplement: S3 Table — (DOCX) [file pgph.0004896.s005.docx]

**S3 Table. Sensitivity analysis-penalized regression**

| **Models** | **Variable** | **Categories** | **aOR (95% CI)** | **P-Value** |
| --- | --- | --- | --- | --- |
| **Maternal factors (N=918)** | ART Regimen | DTG | Ref |  |
|  |  | EFV | 2.36(0.76-7.20) | 0.137 |
|  |  | PI | 26.74(3.61-198.14) | **0.001** |
|  |  | NVP | 1.00(0.05-18.79) | 0.999 |
|  | Duration on ART (years) | >1 | 4.64(0.84-25.48) | 0.077 |
|  |  | 1-2 | 1.37(0.25-7.65) | 0.718 |
|  |  | >2-5 | Ref |  |
|  |  | >5 | 0.60(0.19-1.87) | 0.380 |
|  | Viral load suppression | Suppressed | Ref |  |
|  |  | Unsuppressed | 4.77(1.49-15.25) | **0.008** |
|  | ART history of transition | 0 | Ref |  |
|  |  | 1 | 0.36(0.01-9.12) | 0.534 |
|  |  | 2 | 0.56(0.01-1.90) | 0.146 |
|  |  | 3 | 15.25(0.26-905.73) | 0.191 |
|  |  | 4 | 0.91(0.15-5.62) | 0.920 |
|  |  | 5 | 1 | - |
|  |  | 6 | 1 | - |
|  |  | 7 | 1 | - |
| **Infant factors (N=962)** | Received NVP syrup | Yes | Ref |  |
|  |  | No/Not sure | 1.25(0.44-3.60) | 0.675 |
|  | Received Cotrimoxazole prophylaxis | Yes | Ref |  |
|  |  | No/not sure | 1.00(0.21-4.70) | 0.999 |
|  | Infant Feeding option at the time of first test | Exclusive Breast feeding | Ref |  |
|  |  | Mixed feeding | 25.60(6.11-107.21) | **<0.001** |
|  |  | Not breast feeding | 81.07(17.92-366.73) | **<0.001** |
|  |  | Replacement feeding | 9.77(1.40-68.11) | 0.021 |
|  |  | Unknown | 234.50(60.93-902.45) | **<0.001** |
