# Supplementary material for: Infant HIV-protection: Comparing antiretroviral therapy, maternal and infant factors influence on infant HIV acquisition in Uganda: A six-year real-world experience
Source: PLOS Glob Public Health. 2026 Feb 13;6(2):e0004896. doi: 10.1371/journal.pgph.0004896 (PMC12904416; doi:10.1371/journal.pgph.0004896)
Supplement: Attachment — Submitted filename: Response to Reviewers Comments.docx [file pgph.0004896.s008.docx]

**Response to Reviewers Comments**

We appreciate the Editorial and reviewer’s valuable feedback and have addressed the comments to improve clarity, presentation, and logical flow in the manuscript as follows in this point by point response.

| **Comments** | **Response** |
| --- | --- |
| **Editorial comments** |  |
| 1. Please send a completed 'Competing Interests' statement, including any COIs declared by your co-authors. If you have no competing interests to declare, please state "The authors have declared that no competing interests exist". Otherwise please declare all competing interests beginning with the statement "I have read the journal's policy and the authors of this manuscript have the following competing interests:" | We have revised COI statement to read :The authors have declared that no competing interests exist |
| 2. Please amend your detailed Financial Disclosure statement. This is published with the article. It must therefore be completed in full sentences and contain the exact wording you wish to be published. | This work was supported by the National Institute for Health and Care Research (NIHR) through the Royal Society for Tropical Medicine and Hygiene Early Career Grants Programme (to CA). |
| 3. In the online submission form, you indicated that “The data sets for this study are available upon reasonable request from the corresponding author.” | The data availability statement has been revised. All data used for this manuscript are now included within the manuscript itself and the supplementary materials. |
| 4. If the reviewer comments include a recommendation to cite specific previously published works, please review and evaluate these publications to determine whether they are relevant and should be cited. There is no requirement to cite these works unless the editor has indicated otherwise. | Not applicable |
| Additional Editor Comments:  Thank you for the submission. Kindly address all points raised by the reviewers. In addition to the reviewer points, please ensure that the unit of observation in the study is clearly defined and inclusion criteria are clear. Please include defnitions of terms such as suppressed or non-suppressed viral load, WHO stages etc. In the discussion and limitations kindly discuss unmeasured factors that may confound results. Please comment on completeness of data and how missing data were handled. Please consider collinearity of variables included in the models e.g. ART regimen and duration, NVP prophylaxis and cotrimoxazole prophylaxis, as well as possible interactions. | All points have been addressed in the manuscript: the unit of observation and inclusion criteria are detailed in the Methods, definitions of viral load and WHO stages are provided, completeness of data and missing data handling are described, collinearity and interactions are assessed in the Statistical Analysis Plan, and unmeasured confounders are discussed in the Strengths and Limitations sections. |
| **Reviewer 1** |  |
| 1. For multivariable inclusion in your study, you noted a p >0.2 was included. Please could you give some feedback as to why that cut off was used? | We appreciate the reviewer’s observation. The inclusion criterion was actually p < 0.2 in the univariable analysis, not p > 0.2. This threshold was selected to ensure that potentially important predictors were not excluded prematurely from the multivariable model, acknowledging that some variables may become significant only after adjusting for confounders. |
| 2. There was quite a bit of time spent on the differences in outcomes between maternal age categories. Please could you describe why the ages were split into those specific ranges for the categorical variables and if you feel there is a specific characteristic unique to the age group selected (which is quite broad) that could account for the outcomes observed? | Age was categorized as 15–24, 25–34, and >35 years based on biological relevance and prior research, reflecting differences in experience, adherence, and age-related health risks. Details are provided in the Methods section. |
| 3. I understand that this was not done in the study but while it was meaningful to see the impact infant-related factors had on outcomes relative to maternal-related factors, it would be interesting to see if there is congruence or a relationship between the negative maternal factors and the negative infant factors which could have ended up compounding the subsequent effect or if the two categories (maternal and infant) truly were independent of each other. | Negative infant factors, such as feeding practice, and negative maternal factors, including age above 35 years and viral non-suppression, were explored using descriptive statistics to assess whether they compounded the likelihood of an HIV-positive outcome under PMTCT. The graphical representation in the supplementary material illustrates how these factors interact to influence infant outcomes.  Table 1 further highlights the influence of maternal ART regimens on infant outcomes, providing additional context on how maternal clinical profiles relate to an HIV positive infant status. |
| **Reviewer 2** |  |
| Title- I think experience is preferable to perspective. Perspective had to do with how you see things or a point of view. But this is primary data analysis and think should be objective? | The title has been revised to read **“**Infant HIV-protection: Comparing Antiretroviral therapy, Maternal and Infant Factors Influence on Infant HIV acquisition in Uganda: A six-year Real-World Experience”. |
| Abstract, |  |
| • Line 30: include dates of data collection and inclusion criteria | We have included the study period 2018-2023 and highlighted that included infants with documented HIV status outcomes |
| • Line 31- 32: infant diagnosis is PCR unless they are no longer breastfeeding. How old were this babies and at what point were the babies enrolled into the study? if diagnosis was PCR why call it serostatus. Maybe just HIV status | Thanks for the insight, we have replaced sero status with HIV status or HIV positivity as appropriate throughout the manuscript. |
| • Line 32- 33: descriptive statistics do not summarize the data; they describe the population included in your study | We have revised this statement to read; Descriptive statistics highlighted population characteristics |
| • Line 34: predictors of what? | Bivariate and multivariable logistic regression identified predictors infant HIV status |
| • Line 37: describe the mother-infant pairs where there was transmission a bit more. Eg How old were the infants when final diagnosis was made | Thank you for the insightful comment. Our primary objective was to illustrate maternal and infant factors influencing infant HIV status. We have now added supplementary tables showing the time points at which infants were diagnosed HIV positive, as well as a table highlighting the maternal characteristics associated with HIV-positive infants. |
| • Lines 43- 48: because the prevalence is very low, consider using a Poisson regression with robust error variance | Given that the PMTCT outcome was binary (HIV positive Yes/NO), logistic regression was employed to estimate the odds of an HIV positive outcome associated with receipt of NVP, CTX, and feeding status. We had previously thought of the same approach but the Logistic regression the model provided a better fit (Pseudo R² = 0.51 vs 0.39 Poisson) and generated results that are directly comparable with existing PMTCT literature, which predominantly reports odds ratios. Although Poisson regression can approximate relative risks, the logistic model was more stable and appropriate for this binary outcome. |
| Introduction |  |
| • Line 56: HIV what? Incidence/ new infections, mortality, prevalence? | We have revised to **“**Globally, Mother-to-child transmission (MTCT) remains a major source of paediatric HIV infection” |
| • Line 64- 65: high viral load throughout pregnancy, delivery or breastfeeding is a key driver. Especially in the post-partum period | Thanks you, this statements has been adopted |
| Methods |  |
| • Line 90: the study design should be retrospective cohort study based medical record or chart review. If the design was a retrospective cohort, was there a reason why survival analysis techniques were not used | The phrase under the study design section has been revised to: “This was a retrospective cohort study that reviewed medical records.”  We did not perform survival analysis because the primary objective was not to assess time-to-event outcomes but rather to examine how maternal and infant factors influenced infant HIV status. However, we acknowledge the value of survival analysis and plan to explore its application in future studies to further assess timing and risk of transmission. |
| • Line 94- 95: in the chart (which is not labelled) the authors state the study population was infants with a final result + maternal information available but hear it says infants and pregnant/ BF mothers Be consistent with the terms | The figure has now been labeled. As the data collection process.  The statement has been rephrased for clarity as follows:  Total infants included were all infants with a final result (Infants with mother infant pairs (918) and infants without mother pairs (44)) = 962. |
| • Line 96: the relevant Ugandan PMTCT guidelines should be summarized here or included as a supplementary file | The PMTCT guidelines are include in the Uganda consolidated guidelines for HIV care and treatment in Uganda which have been referenced in our work as reference number 2. We added the testing algorithms in the supplementary material |
| • Line 97: were there other inclusion criteria eg infants born in a given period and followed up until a given time | No, we included all the infants in the stsudy period with documented HIV stausts between 2018-2023. |
| • Line 109: the inclusion criteria in the middle box/ tier should be included with others in the section on study population., Who are the participants - infants? | The word participants has been rectified to clearly state infants throughout the flow chart |
| • Line 115: regarding Stata 16 add manufacturer and city | The software has been updated to include manufacturer and city. The revised text now reads:  **Data were analyzed using Stata version 16 (StataCorp, College Station, Texas, USA).** |
| • Line 116: regarding infant seroconversion, babies are born seropositive from exposure to maternal antibodies. Depending on age, they may not seroconvert but remain seropositive when infected. Please add in the setting section how infant testing is done in Uganda | This was an oversight. All references to seroconversion and serostatus have been replaced with HIV status. HIV diagnosis in Uganda is conducted using DNA PCR for infants under 18 months and serology for infants aged 18–24 months. This information has been added to the study setting section for clarity. |
| Results |  |
| • Line 136: please describe the infants better before reporting on those who were HIV positive at the final diagnosis | Thank you for the insight. We combined infant and maternal characteristics with infant outcomes in Table 2 (now Table 1) to streamline presentation and avoid redundancy. All relevant demographic and clinical characteristics are included to provide sufficient context before reporting HIV status at final diagnosis.  To improve perspective and logical flow, we have switched the positions of the two tables: Table 1 now presents infant and maternal outcomes, while Table 2 shows the influence of PMTCT ART regimen on infant HIV positivity. |
| • Table 2 lists under maternal characteristics viral suppression. At what time point since viral load is a time varying variable | Thank you for the observation. The viral load suppression reported under maternal characteristics in Table 2 refers to viral load measured during pregnancy. This qualifier has been added in table two as well. |
| • Table 3: there were only 18 babies that were HIV positive. The authors shouldn’t have more than two variables their models. Also data collection methods should include how variables were selected in the multivariate model | Thank you for the observation. We acknowledge that with only 18 HIV-positive infants, including too many variables in the multivariate model could lead to overfitting. To address this, we limited the model to key variables with strong biological plausibility and demonstrated associations in univariate analysis.  Additionally, the evaluated variables for multivariable model were selected based on significance in univariate analysis of p value < 0.2 as stated in the statistical analysis plan. |
| • Table 3: the authors should consider a Poisson regression with robust error variance | Given that the PMTCT outcome was binary (HIV positive Yes/NO), logistic regression was employed to estimate the odds of an HIV positive outcome associated with receipt of NVP, CTX, and feeding status. We had previously thought of the same approach but the Logistic regression the model provided a better fit (Pseudo R² = 0.51 vs 0.39 Poisson) and generated results that are directly comparable with existing PMTCT literature, which predominantly reports odds ratios. Although Poisson regression can approximate relative risks, the logistic model was more stable and appropriate for this binary outcome. |
| • Table 3: ART history of transition was not defined in the table footer | Thank you for the observation. A footer has been added to table 3 |
| • Line 173: why compare the two models. It’s not either/ or. Did you try to build a model that combines both maternal and infant factors in one model | Thank you for the insight. The primary focus of the study was to identify which factors influence HIV-positive outcomes in order to guide where HIV prevention strategies should be prioritized i.e. whether maternal or infant. |
| Discussion |  |
| • Line 196: most studies show higher MTCT risk with younger mothers. Can you explain reasons why this is not the case in your study? What % of babies had a final result? Did you compare babies with a final result and those without? Do these babies differ by maternal age? Please compare include a table before ta | This finding may reflect HIV program risk stratification, as younger mothers are often perceived as higher risk and thus receive enhanced support within PMTCT programs, while older mothers may receive relatively less intensive intervention.  Infants without a final results were not included in this study so there were no such a comparison. |
| • Line 239: the conclusion not to scale up PI based regimens in pregnancy doesn’t make sense. They are mostly used as third line or first or second line when DTG or EFV cannot be used. If the PIs need to be used what then is the recommendation | The statement has been revised to read; Our findings highlight the importance of scaling up all ART regimens in PMTCT programs, except PI-based regimens, unless DTG-, EFV-, or NVP-based regimens are contraindicated or have documented resistance. |
