# Supplementary material for: Infant HIV-protection: Comparing antiretroviral therapy, maternal and infant factors influence on infant HIV acquisition in Uganda: A six-year real-world experience
Source: PLOS Glob Public Health. 2026 Feb 13;6(2):e0004896. doi: 10.1371/journal.pgph.0004896 (PMC12904416; doi:10.1371/journal.pgph.0004896)
Supplement: Attachment — Submitted filename: Response to Reviewers2.docx [file pgph.0004896.s009.docx]

**Response to Reviewers**

We appreciate the reviewers’ valuable feedback and constructive comments, which have significantly strengthened our manuscript. In response, we have carefully revised the document to address all suggestions and improve clarity, organization, and methodological precision. Below, we provide a detailed summary of the changes made in line with Editorial and reviewer’s comments.

| **Comments** | **Response** |
| --- | --- |
| **Editorial comments** |  |
| 1. If the reviewer comments include a recommendation to cite specific previously published works, please review and evaluate these publications to determine whether they are relevant and should be cited. There is no requirement to cite these works unless the editor has indicated otherwise. | This is not applicable in our case, as no specific publications were recommended for citation by the reviewers. |
| 2. Please review your reference list to ensure that it is complete and correct. If you have cited papers that have been retracted, please include the rationale for doing so in the manuscript text, or remove these references and replace them with relevant current references. Any changes to the reference list should be mentioned in the rebuttal letter that accompanies your revised manuscript. If you need to cite a retracted article, indicate the article’s retracted status in the References list and also include a citation and full reference for the retraction notice. | We have thoroughly reviewed our reference list to ensure completeness and accuracy. |
| While revising your submission, we strongly recommend that you use PLOS’s NAAS tool (https://ngplosjournals.pagemajik.ai/artanalysis) to test your figure files. NAAS can convert your figure files to the TIFF file type and meet basic requirements (such as print size, resolution), or provide you with a report on issues that do not meet our requirements and that NAAS cannot fix. | All figures were reformatted using the PLOS NAAS tool and now meet the journal’s specifications. |
| **Reviewer comments** |  |
| Thank you for the opportunity to review the revised manuscript. It reads better than the previous version. The authors have addressed most comments but there are others that have not been addressed. I have listed the issue that need to be resolved below | We appreciate the time and effort the reviewer has dedicated to carefully evaluating our revised manuscript. |
| - the abstract on the system and the one in the text are not the same. I don't know which one is correct | This was an oversight on our side. We have now ensured that all abstracts are consistent, with the version in the main text being the correct one. |
| - although the authors changed the population to infants, the paper is still written like the mothers are the study population. eg | We have revised the statement to ensure that the infants are clearly presented as the study population, as shown below |
| i) Line 90-study design says study of medical records PLHIV attending PMTCT, | Thank you for the insight. The statement has been revised to read:  “We reviewed medical records of HIV-exposed infants enrolled in the Early Infant Diagnosis (EID) clinic at Mildmay Uganda Hospital (MUgH).” (Lines 30 and 88) |
| ii) line 99- start with infant data since the infants are your participants and then describe how matching maternal data was collected, | “Data on infant characteristics, including receipt of nevirapine (NVP) syrup for prophylaxis, receipt of cotrimoxazole prophylaxis, and the infant feeding option at the time of the first HIV test, were obtained from the Early Infant Diagnosis (EID) registers. Corresponding maternal data, including socio-demographic characteristics and medical history, were extracted from the PMTCT registers, patient cards, and Electronic Medical Records (EMR).” (Lines 96-99) |
| iii) Tables 1 and 3 where maternal data is presented. the authors should present infant data first | Data in Tables 1 and 3 have been reorganized to present infant-related variables first, along with their corresponding descriptions. Lines 135-143, 158-168) |
| - in the supplementary tables 1 and 2 shouldn't the denominator for the proportions be the number of HIV positive children and not those tested? | Thank you for this valuable observation. The proportions in Supplementary Tables 1 and 2 have been recalculated using the number of HIV-positive children as the denominator to ensure accuracy and consistency with the analysis. (Supplementary material) |
