# Supplementary material for: Infant HIV-protection: Comparing antiretroviral therapy, maternal and infant factors influence on infant HIV acquisition in Uganda: A six-year real-world experience
Source: PLOS Glob Public Health. 2026 Feb 13;6(2):e0004896. doi: 10.1371/journal.pgph.0004896 (PMC12904416; doi:10.1371/journal.pgph.0004896)
Supplement: Attachment — Submitted filename: Response to comments 11.12.2025.docx [file pgph.0004896.s010.docx]

**Response to comments**

We appreciate the reviewers’ valuable feedback and constructive comments, which have significantly strengthened our manuscript. In response, we have carefully revised the document to address all suggestions and improve clarity, organization, and methodological precision. Below, we provide a detailed summary of the changes made in line with reviewer’s comments.

| **COMMENT** | **RESPONSE** |
| --- | --- |
| Reviewer #2: Thank you for the opportunity to re-review this manuscript with the data included. A few more concerns |  |
| - line 35 still refers to mother-infant pairs when it should be infants | Thanks for this observation, this has been revised to read “HIV positivity among 918 infants with maternal data was 1.96% (18)”  (Line 37) |
| - Line 39 and 40. are these ORs for NVP and CTX adjusted ORs or crude ones. This is because table 3 shows different numbers | The earlier reported Odds ratio were crude and we resorted to reporting only adjusted Odds ratios in the abstract. |
| - Line 165 : the association between remaining on a PI based regimen was not significant in the multivariable model. Please indicate that this is a crude OR | Thanks for this observation, The association between remaining on a PI-based regimen was not significant in the multivariable model. Crude associations are reported as COR, and adjusted associations as aOR throughout the manuscript for clarity ((Lines 167-169) |
| - Table 3: did the univariable and multivariable logistic regression only include infants for whom maternal data was available? if that was the case, the regression model would have 18 outcomes and there only a maximum of two variables included. Its not indicated in the table how many infants were included in the infant model or maternal model | We have reported the number of participants included in each model in Table 3: Maternal predictors (N = 918) and Infant predictors (N = 962).  (Table 3) |
| Reviewer 3 |  |
| Study Design • The retrospective cohort design is appropriate for exploring associations between maternal and infant factors and HIV outcomes in real-world PMTCT settings. Six years of data collection adds temporal breadth. However, no a-priori power calculation or event-per-variable justification is provided. With only 18 positive outcomes (1.96%), the analysis is underpowered. The retrospective nature precludes control for unmeasured confounding. Exclusion criteria were not clearly defined, introducing selection bias. Recommendation: Include a post-hoc power analysis and clearly define exclusion logic. Frame findings as exploratory rather than confirmatory. | Thank you for appreciating the design and duration. We have addressed the missing details for clarity and provided justification where adjustments were not performed, as outlined below.  Post hoc analysis: A post hoc analysis was conducted, estimating power as the proportion of simulations with p < 0.05, which was 83%, indicating adequate sensitivity to detect effects of this magnitude ( Lines 130-135)  Exclusions: Of 976 records, 14 (1.4%) were excluded due to missing HIV status outcome. This low level of missingness is unlikely to materially affect the validity or statistical power of the study. magnitude ( Lines 123-125)  Framing of findings: Given the wide confidence intervals, findings are presented as suggestive rather than confirmatory. |
| Variable Definition and Data Handling • No. clear description of how missing data was handled. No testing of missingness mechanism (MCAR, MAR, MNAR) was performed. | Out of 976 participants, 14 (1.4%) were excluded due to missing HIV status outcome data. Given the very low proportion of missing data, we did not perform formal testing for the missingness mechanism (MCAR, MAR, MNAR), as the impact on study validity and statistical power is expected to be minimal. ( Lines 123-125) |
| • How was adherence measured from retrospective data? | Adherence was measured retrospectively using clinical notes indicating adherence categorization (good, fair, poor), consistent with standard PMTCT program definitions. ( Table 1) |
| • Variable selection for the multivariable model is unclear. `or example the exclusion of maternal age and adherence but the inclusion of history of transition despite evidence from the bivariate/univariate models. • Recommendation: Apply multiple imputation or sensitivity analysis for missingness. Model Specification and Validity | Variables included in the multivariable model were selected based on a combination of biological plausibility, relevance from the literature, and results from univariable analyses.  For age and adherence, these were dropped by model given their perfect prediction of the outcome; hence no output from the model as indicated in table 3 |
| • The term 'hierarchical logistic regression' is misused; no multilevel structure seems to be described or shown. It looks like two independent logistic models were run. | We thank the reviewer for this observation. We agree that the term “hierarchical logistic regression” was not appropriate. We have revised the manuscript to clarify that two separate logistic regression models were fitted independently: one for maternal factors and one for infant factors. ( Lines 112-115) |
| • With 18 HIV-positive cases and >8 covariates, event-per-variable ratio ≈ 2, causing overfitting. A more robust regression model (mixed effects or penalized is preferred). It is not clear how you handled multiple births/ infants from one mother. | Thank you for the comment regarding the analysis. We had earlier conducted a sensitivity analysis using Firth’s penalised logistic regression to mitigate small-sample bias and address potential instability arising from sparse data. The penalised estimates were directionally consistent with the primary logistic regression results and had comparable confidence intervals, supporting the robustness of the observed associations (see supplementary material). In addition, the dataset contained no mother–infant pairs with multiple births; each infant corresponded to a unique mother, and therefore clustering at the maternal level was not present. Furthermore, as this was a single-site study, there was no clustering at the health-facility level; thus, a mixed-effects analysis was not plausible. |
| • No model calibration or discrimination metrics reported. Model Comparison (AIC/BIC)  • AIC/BIC values were compared across models with different sample sizes (821 vs. 962), which invalidates comparability. Interpretation of AIC as model superiority is incorrect. Recommendation: Use likelihood-ratio tests or cross-validated AUCs on identical datasets for valid comparison. | We thank the reviewer for highlighting this point. We acknowledge that AIC and BIC values are not directly comparable across models fitted on different sample sizes; accordingly, they were interpreted descriptively within each model rather than used for cross-model comparison. Model performance was therefore assessed using complementary measures, including pseudo-R² and discrimination metrics. Formal calibration measures were not undertaken due to the very small number of outcome events, which limits their reliability. Discrimination was evaluated using the area under the receiver operating characteristic curve (AUC). For the maternal factors model (n = 821), the standard AUC was 0.74, indicating acceptable discrimination; however, cross-validated AUC was unreliable due to sparse events, so bootstrap resampling was used for internal validation, yielding moderate discrimination (AUC 0.70, bootstrap 95% CI 0.58–0.83). For the infant factors model (n = 962), the standard AUC was 0.94, and the cross-validated AUC was 0.95 (95% CI: 0.79–0.95), indicating excellent discrimination. ( Lines 117-118) |
| Statistical Reporting and Transparency • Extremely wide CIs indicate quasi-complete separation. No pseudo-R² reported. No multiple testing correction. Recommendation: Report Nagelkerke R², apply Bonferroni/FDR corrections, and identify unstable estimates explicitly. | We thank the reviewer for the comment. We acknowledge that some odds ratios had extremely wide confidence intervals, to address this, we report pseudo-R²(Table 4) for all models to indicate explained variance. Multiple testing corrections were not applied because each model evaluated a pre-specified set of variables individually with respect to the outcome, and no multiple comparisons were performed; therefore, the risk of inflated Type I error was minimal. Additionally, sensitivity analyses using Firth’s penalized logistic regression were conducted to reduce small-sample bias and confirm robustness, with penalized estimates directionally consistent with the primary model |
| Rare-Event Bias • Standard logistic regression under rare events yields biased MLE estimates. • Recommendation: Implement rare-event logistic regression or penalized likelihood methods. Also explains the very wide Cis. | We thank the reviewer for this observation. We acknowledge that standard logistic regression can yield biased maximum likelihood estimates in the presence of rare events, which likely contributed to the extremely wide confidence intervals observed for some predictors. To address this, we had earlier conducted sensitivity analyses using Firth’s penalized logistic regression, which reduces small-sample and rare-event bias. The penalised estimates were directionally consistent with the primary models, supporting the robustness of our findings (Supplementary material). |
| Interpretation and Validity of Conclusions • Claims of 'better model fit' are overstated; evidence supports exploratory correlation, not predictive superiority. • Recommendation: Revise language to reflect exploratory, hypothesis-generating interpretation. | We thank the reviewer for this comment. We acknowledge that the analyses were intended to explore associations between maternal and infant factors and HIV transmission, rather than to establish predictive superiority. Accordingly, we have revised the language throughout the manuscript to emphasize the exploratory and hypothesis-generating nature of the findings. |
| Reviewer Summary Statement The study is conceptually valuable and clinically relevant but suffers from significant statistical fragility due to rare events, over-fitting, and improper model comparison. Re-analysis with penalized regression, improved model diagnostics, and reproducible code is required before acceptance for publication. | We thank the reviewer for this constructive critique. Given the rarity of events, all multivariable analyses were re-estimated using penalized (Firth) logistic regression to reevaluate the consistence of findings in the standard logistic regression. Model performance was reassessed using discrimination metrics (AUC with 95% confidence intervals). All the codes used for this analysis have been provided as supplementary material. |
