# Supplementary material for: Infant HIV-protection: Comparing antiretroviral therapy, maternal and infant factors influence on infant HIV acquisition in Uganda: A six-year real-world experience
Source: PLOS Glob Public Health. 2026 Feb 13;6(2):e0004896. doi: 10.1371/journal.pgph.0004896 (PMC12904416; doi:10.1371/journal.pgph.0004896)
Supplement: Attachment — Submitted filename: Response to Reviewers.docx [file pgph.0004896.s011.docx]

**Response to Reviewers**

We appreciate the feedback and constructive comments, which have significantly strengthened our manuscript. In response, we have carefully revised the document to address all suggestions and improve clarity, organization, and methodological precision. Below, we provide a detailed summary of the changes made in line with the given comments.

| **Comment** | **Response** |
| --- | --- |
| Thank you for the revision. No responses were received for the following two points. Kindly address before acceptance for publication:  1. Please note that the terminology of MTCT (mother-to-child-transmission) is changing to vertical transmission (VT) so that language is more inclusive and less stigmatising. Kindly edit the paper accordingly. | Thank you for highlighting this point. We have revised the manuscript to replace the terminology mother-to-child transmission (MTCT) with vertical transmission (VT) throughout the text. In addition, all references to prevention of mother-to-child transmission (PMTCT) have been updated to prevention of vertical transmission (PVT), in line with current, more inclusive and less stigmatizing terminology.  However, given that prevention of mother-to-child transmission (PMTCT) remains the most commonly used term in the existing literature, contexts and indexing systems, we request permission to include it among the keywords to enhance discoverability. The term has not been included in the manuscript text. |
| 2. Please also ensure the paper undergoes careful editing for grammatical, formatting and spelling errors including missing words, repeated words and inconsistent font which were identified on review. | We have carefully proofread the entire manuscript to address grammatical, spelling, and formatting issues, including missing or repeated words and inconsistent font usage. |
